# The West Texas Medical Association

**Published:** 1902-11

**Authors:** 


### The West Texas Medical Association.

The West Texas Medical Association's twenty-sixth annual meeting, held in San Antonio on the morning, afternoon and evening of Thursday, October 23rd, in point of attendance and enthusiasm easily eclipsed any gathering of the kind (except the meetings of the State Association) ever held in Southwest Texas.

The morning session was called to order by President W. E. Luter at 9:30 o'clock, and the following program carried out:

Paper, "Diseases in the Tropics," by Major Charles F. Mason, U. S. A. This paper, being based upon the experience of a medical officer during his service in Porto Rico and in the Philippine Islands, contained much valuable data tending to show the modifying influences exerted upon many diseases by tropical climates. Dr. Mason takes the ground that there are, strictly speaking, no "tropical diseases." Diseases as they occur in the tropics are, however, greatly modified by climate, environment, and habit.

Dr. T. T. Jackson, of San Antonio, who has also seen service in the Philippines, led the discussion. He was impressed by the remarkably rapid and fatal course pursued by pulmonary tuberculosis, both among the natives and the soldiers. He observed that after the disease manifested itself the patient rarely ever survived more than a few weeks. Even when started for home as soon as the diagnosis was made few lived to reach their destination. It is his opinion that two-thirds of all the men stationed in the Philippines suffer from intestinal parasites, while practically all of the natives suffer in this manner. Santonin tablets are kept at all of the shops and the natives eat them as they would candy. As for amebic dysentery, he does not believe it to be curable in a tropical climate.

Dr. B. F. Kinglsey, of San Antonio, agreed fully with Dr. Mason that environment and habit, rather than climate, are responsible for the occurrence of many diseases which are commonly met with in the tropics.

Dr. T. A. Williams, of Edinburg, Scotland, late of Cape Colony, called attention to the fact that certain observers had demonstrated the larva of the *ankylostoma duodenale* in the skin of patients suffering from the so-called "Dohbie itch." During Dr. Williams's residence in South Africa, he had confirmed the observations of Dr. Brodie as to the frequent occurrence of a peculiar intractable catarrhal rhinitis in Johannesburg and vicinity. The discharge from the nostrils of these patients had been found to contain the pneumococcus of Fränkel. It is interesting to note that at the same time croupous pneumonia occurs with great frequency and with an exceedingly high mortality rate in that neighborhood. With reference to the non-curability of tuberculosis in the Philippines referred to by Drs. Mason and Jackson, he believed that not the climate, but bad sanitary conditions, especially the crowding together of patients, is responsible for the prevalence of the disease and its resistance to treatment. He believed, with Valther, of Nordrech, in Germany, that 90 per cent. of cases of tuberculosis taken in the early stages and properly managed can be cured.

Dr. F. Paschal, of San Antonio, agreed with the previous speakers that not climate, but poor sanitary conditions and vicious habits are responsible in all localities for the prevalence of zymotic diseases and of the high mortality rate resulting therefrom. To illustrate, he related the following:

"Some years ago, a band of Indians that had been committing depredations in Mexico was captured and all of the adults, male and female, killed. The 250 children were taken prisoners and brought to Chihuahua, Mexico, where they were taken by families as servants. Being removed from the freedom of out-door life, and placed under the worst possible sanitary conditions, every one of the number died in less than five years, tuberculosis being responsible for most of the deaths."

Dr. H. A. West, of Galveston, said that the prevalence of intestinal diseases and especially of amebic dysentery in the Philippines and their frequent association with hepatic abscess is of great interest to us in this semi-tropical climate. Neither he nor Dr. W. M. Wolff, of San Antonio, who followed him in the discussion, could agree with Dr. Jackson in saying that amebic dysentery is an incurable disease; both had seen cases showing all the classical symptoms and the diagnosis of which had been confirmed by the microscope, recover full under proper treatment and surroundings.

Paper, "The Radical Treatment of Mastoiditis, with Report of Cases," by Dr. Robt. E. Moss, of San Antonio. The writer said that specialists are as much divided as to when to operate in cases of mastoiditis as are general surgeons as to when to operate in cases of appendicitis. Personally he believed that a radical operation should be done in every well-established case, and the time limit after the first appearance of the symptoms in the average case should not exceed five or six days. Every case of chronic or recurring mastoiditis should be operated upon, as should also every case following scarlet fever. The operation should be thorough. He had often regretted having done too little, but has never regretted having done too much. The doctor then described the steps of the operation, and followed this with a report of several interesting cases.

Dr. J. H. Burleson, of San Antonio, led the discussion and agreed with Dr. Moss that in the majority of cases palliative treatment is not to be even thought of.

Dr. J. V. Spring stated his belief that many cases could be successfully managed without resort to an operation.

Drs. A. C. McDaniel and F. Paschal, of San Antonio; Dr. B. E. Hadra, of Dallas, and Dr. T. A. Williams, of Edinburg. Scotland, also took part in the discussion.

Paper, "Murphy's Method of Lung Compression as a Treatment for Pulmonary Tuberculosis," by Dr. Thos. P. Lloyd, of San Antonio. This paper was illustrated by the apparatus for compressing the lungs by means of the injection of nitrogen into the pleural cavity. Dr. Lloyd explained that the treatment was not applicable in cases with extensive adhesions existing, nor could it in any sense be considered a "cure-all." It is, however, in many cases a valuable adjunct to other means of treatment.

Dr. H. J. Chapman, of San Antonio, stated that while he was not entirely familiar with this method of treatment, he believed that it deserved a careful trial by the profession. He had been much impressed by the favorable reports made by those who had tried it in their practice. In cases with cavity formation it will no doubt assist the healing process.

Dr. C. E. R. King, of San Antonio, stated that he did not believe that lung compression is ever advisable under any circumstances.

Dr. F. Paschal, of San Antonio, took much the same view.

Dr. Hadra, of Dallas, and Dr. Jackson, of San Antonio, expressed themselves as being in favor of giving the treatment a trial.

The afternoon session opened with a clinic at which the following cases were presented:

Four cases of general paresis by Dr. G. H. Moody, of the Southwestern Insane Asylum; a case of general paresis and a case of multiple sclerosis, by Dr. M. L. Gravas, Superintendent of the Southwestern Insane Asylum; a case of lateral curvature of the spine, by Dr. F. Paschal, of San Antonio. Dr. Paschal also exhibited a patient who had been a sufferer from stricture of the urethra with urinary extravasation and upon whom he had done a combined suprapubic cystotomy and perineal urethrotomy. These cases were extremely interesting and were generally discussed.

Paper, "The Treatment of Croupous Pneumonia," by Dr. H. A. West, of Galveston. Dr. West outlined the treatment that he had found to be best in the treatment of this disease. He denounced the use of the coal tar derivatives. The paper was well received and developed a considerable amount of discussion, in which the following members and visitors participated: Drs. F. Paschal and M. M. Edmonson, of San Antonio; Dr. H. A. Tutwiler, of Flatonia; Dr. J. M. Frazier, of Belton, and Dr. Geo. R. Tabor, of Austin.

Address, "The Social Aspect of Tuberculosis," by Dr. F. E. Daniel, of Austin. Dr. Daniel spoke without notes and treated his subject in a most interesting and entertaining manner. At the close of his address, the association extended a vote of thanks to the speaker, and on motion of Dr. F. Paschal, of San Antonio, seconded by a dozen voices at once, a resolution was unanimously adopted providing for the reproduction of Dr. Daniel's speech in the daily press.

At the evening session the following officers were elected for the ensuing year: President, Dr. T. T. Jackson, San Antonio; Vice-Presidents, Dr. B. M. Grace, Seguin, and Dr. W. M. Wolff, San Antonio; Secretary and Treasurer, Dr. W. B. Russ, San Antonio; Board of Censors, Drs. W. E. Luter, F. Paschal, C. E. R. King, B. F. Kingsley and J. H. Burleson, all of San Antonio.

The members of the Austin District Medical Society extended, through Drs. M. M. Smith and W. A. Harper, an invitation to the members of the West Texas Medical Association to meet with them at their annual meeting in December. Their invitation was accepted by a unanimous vote. Invitations were also received by the Association to meet during the coming year at Seguin, through Dr. Frazier on behalf of the Central Texas Medical Society, and to meet at Marlin. Both these were also accepted.

The Association then adjourned to attend the annual banquet held at Hotel Menger, where covers were laid for fifty. The following menu was served:

A Manhattan a la Shropshire.
Shell oysters a la Heaney. Celery.
Daniel's cream of tomatoes.
*Haut Sauterne.*
Fresh lobsters de Burg. Wilson's olives.
Braized sweetbreads, Taborized. King's English peas.
*Pontet Canet.*
Filets of tenderloin, Kingsley style.
Mossy mushrooms. Duchess potatoes.
Roast turkey a la West. Asparagus.
*Mumm's Extra Dry.*
Watt's salad.
Vanilla ice cream au Paschal. Hick's assorted cakes.
Jacksonian Roquefort cheese. Smith's Edam cheese.
Clear's coffee with milk au Campbell.
Cigars.

During the evening the following toasts were offered and responded to, Dr. C. E. R. King acting as toastmaster:

"The Governor," Dr. Geo. R. Tabor, Austin.

"Reorganization of the State Medical Association," Dr. Walter Shropshire, Yoakum.

"Rudolf Virchow," Dr. Sigmund Burg, San Antonio.

"Our Departed Brothers," Dr. F. E. Daniel, Austin.

"Our Guests," Dr. Robert Moss, San Antonio.

"The Texas State Medical Association," Dr. H. A. West, Galveston.

"The State Board of Medical Examiners," Dr. M. M. Smith, Austin.

"The Medical Corps U. S. Army," Capt. T. S. Bratton, U. S. A., Fort Sam Houston.

"The Volunteer Surgeons, U. S. Army." Dr. T. T. Jackson, San Antonio.

"The Relation of the Medical Profession and Public Education," Dr. John S. Lankford, San Antonio.

"The Relation of the Medical Profession to the Public Health," Dr. Frank Paschal, San Antonio.

"The Eleemosynary Institutions of the State," Dr. Marvin L. Graves, Southwestern Insane Asylum.

"The Ladies," Dr. L. L. Shropshire, San Antonio.

"The Young Men in the Profession," Dr. Thos. P. Lloyd, San Antonio.

"Our Sister Societies," Dr. W. A. Harper, Austin.

Major Charles F. Mason, U. S. A., proposed a toast to "The President." This was drunk standing and in silence.

The following visitors and members were present at the meeting: B. E. Hadra, Dallas; H. Daroch, Fredericksburg; J. M. Frazier, Belton; T. A. Williams, Edinburgh, Scotland; L. Hirschfield, Marion; M. B. Grace, Seguin; Geo. R. Tabor, Austin; M. M. Smith, Austin; W. A. Harper, Austin; F. E. Daniel, Austin; Capt. T. S. Bratton, U. S. A., Ft. Sam Houston; Maj. Chas. F. Mason, U. S. A., Ft. Sam Houston; H. A. West, Galveston; Walter Shropshire, Yoakum; A. Garwood, New Braunfels; H. A. Tutwiler, Flatonia; W. T. Rape, Ballinger; H. Leonards, New Braunfels; J. M. Hons, San Marcos; Ernest Palmer, Kerrville; R. H. Harrison, Columbus; W. T. Reeve, Fisher's Store. From San Antonio: P. Baldessarelli, James Bartlett, H. D. Barnitz, D. Berrey, H. A. Blair, A. A. Brown, J. H. Burleson, S. Burg, H. J. Chapman, E. C. Clavin, C. M. Decker, A. D. Dupuy, M. L. Graves, F. M. Hicks, E. T. Hughes, T. T. Jackson, J. W. Kenney, C. E. R. King, B. F. Kingsley, J. S. Lankford, T. J. Largen, T. P. Lloyd, W. E. Luter, A. S. McDaniels, T. E. Moore, Robt. E. Moss, J. P. Oldham, F. Paschal, R. Robinson, W. B. Russ, J. V. Spring, L. L. Shropshire, G. G. Watts, W. M. Wolff, F. A. Davis, R. L. Withers, E. H. Elmendorf, J. H. Moore, G. H. Moody, R. A. Goeth, M. M. Edmonson, J. F. Hines. Total in attendance, 65.

W. B. Russ, Secretary.

